# Toxicology

**Published:** 1837-01

**Authors:** 


### TOXICOLOGY.

*Deleterious Effects of Pork.* By J. M'DIVITT, M.D., Canterbury.

IT is commonly known that fresh pork, to those unused to it, is apt to produce diarrhœa and griping pains; but Dr. M'Divitt has met with several cases in which the abdominal symptoms were equal in severity to those produced by acrid poisons.

CASE I. A man was seized, seven hours after eating salt pork, with acute burning pain in the epigastric and umbilical regions, and constant vomiting; pulse weak, extremities cold; cold perspiration covered the face; he complained of a dry burning sensation in his throat. Poisoning was suspected: an emetic was given, and some lumps of half-digested meat expelled, with relief to the pain in the epigastrium; the bowels acted, and the pain round the umbilicus was then removed. The next morning he went to work.

CASE II. A hale man was attacked, about three P.M., with pain, like cramp in the stomach; his respiratory muscles seemed spasmodically fixed; countenance pale and anxious; pulse feeble and intermittent; hiccup, followed by an ineffectual attempt at vomiting. He complained that something lay at his stomach, hard and heavy as a stone. Two hours before he had dined on fried pork. An emetic was given, several pieces of pig's liver and fat pork brought up, and he felt free from pain.

CASE III. Similar to the others. The pain was situated over the duodenum, and aggravated by the slightest pressure.

CASE IV. A robust young man dined on pork, about two in the afternoon: seven hours afterwards he was attacked with agonizing pain in the abdomen and vomiting. The next morning the pain was still violent, with some vomiting. He had been purged several times, with temporary relief; abdomen slightly swollen, and very tender; face and eyelids swollen; breast, arms, and legs covered with urticaria; pulse quick and sharp. An emetic and purgative were given: the pain and swelling of the abdomen were removed the next day, but the eruption appeared and disappeared by turns for a week, and was only cured by purging.

Two other cases are given: in one, the pain came on twenty-three hours after the pork was eaten, and Dr. M'Divitt says it was situated in the sigmoid flexure of the colon: the other case is imperfectly reported, as there is nothing said to prove that pork had been taken. Several other cases have occurred in Dr. M'Divitt's practice. In every case *fat* pork had been eaten, but no alteration could be detected in its sensible properties: in some instances several persons had eaten of the same pork, and only one was affected; and this person has in general repeatedly eaten pork, and even from the same pig, without injury.

*Edinburgh Medical and Surgical Journal, October*, 1836.

